# A comprehensive bioinformatic evaluation of the NTRK family’s potential as prognostic biomarkers in breast cancer

**DOI:** 10.1093/bioadv/vbaf030

**Published:** 2025-02-21

**Authors:** Ramtin Mohammadi, Mohsen Ghiasi, Saber Mehdizadeh, Javad Mohammadi, Shahla Mohammad Ganji

**Affiliations:** Department of Molecular Medicine, Medical Biotechnology Institute, National Institute of Genetic Engineering and Biotechnology (NIGEB), Tehran, 1497716316, Iran; Medical Biotechnology and Bioinformatics Research Group (MBBRG), Universal Scientific Education and Research Network (USERN), Tehran, 14197331, Iran; Rajaie Cardiovascular Medical and Research Center, Iran University of Medical Sciences, Tehran, 1995614331, Iran; Department of Immunology, School of Medicine, Mazandaran University of Medical Sciences, Sari, 4815733971, Iran; Department of Molecular Medicine, Medical Biotechnology Institute, National Institute of Genetic Engineering and Biotechnology (NIGEB), Tehran, 1497716316, Iran; Department of Life Science Engineering, Faculty of New Sciences and Technologies, University of Tehran, Tehran, 1439957131, Iran; Department of Molecular Medicine, Medical Biotechnology Institute, National Institute of Genetic Engineering and Biotechnology (NIGEB), Tehran, 1497716316, Iran

## Abstract

**Motivation:**

Breast cancer (BC), with its rising prevalence and mortality rate, is one of the most significant human health issues. The family of transmembrane tyrosine kinases that promote neuronal growth includes the neurotrophic tyrosine kinase receptors (NTRKs). NTRK1–3 genes encode the members of this family. Alterations of NTRK genes can induce carcinogenesis both in neurogenic and non-neurogenic cells. The prevalence of NTRK gene fusion is under 1% in solid tumours but is highly encountered in rare tumours. Since the prognostic values of NTRK families’ expression in various types of cancer are becoming increasingly evident, we aimed to conduct a comprehensive bioinformatics study evaluating the prognostic significance of the NTRK family in BC. Online bioinformatic databases including TCGA, UALCAN, Kaplan–Meier plotter, bc-GenExMiner, cBioPortal, STRING, Enrichr, and TIMER were utilized for analysis.

**Results:**

High levels of NTRK2 and 3 demonstrated better associations with overall survival (OS) and recurrence-free survival (RFS) in BC patients (*P* < .05), while high levels of NTRK1 showed an applicable correlation with RFS in BC patients (*P* < .001). Our findings provide a new outlook that might aid in the field of personalized medicine and therapeutic use of NTRK as a prognostic biomarker in BC.

**Availability and implementation:**

All data generated or analysed during this study are included in this published article.

## 1 Introduction

Breast cancer (BC) is the most prevalent cancer and the main reason for cancer-related deaths among females worldwide (Łukasiewicz *et al.* 2021, Alizamir *et al.* 2022, Tzenios *et al.* 2024). BC is the primary cause of cancer-related mortality globally, accounting for about 665 000 fatalities (about 16%, or one in every six cancer-related deaths) among women (Arnold *et al.* 2022, Bray *et al.* 2024). Despite the fact that the most developed regions of the world have the highest relative incidence of BC, over half of all cases are diagnosed in low- and middle-income nations due to the huge populations in less developed regions, posing a major disease burden (Wilkinson and Gathani 2022). BC is a complex illness with substantial heterogeneity both within and between tumours (Fumagalli and Barberis 2021). According to molecular features, BC is classified into five intrinsic subtypes, including Luminal A, Luminal B, Human Epidermal growth factor Receptor 2 (HER2)-enriched, Basal-like, and Normal-like (Johnson *et al.* 2021, Rossing *et al.* 2021, Berg *et al.* 2024). The development of therapeutic and diagnostic approaches demonstrates the intricate complexity of BC subtypes, and it is expected that in the future, more definite and exact descriptions based on novel factors (such as related genes) will be discovered.

A family of transmembrane receptor tyrosine kinases known as neurotrophic tyrosine receptor kinase (NTRK) genes is crucial for the development of the nervous system (Hechtman 2022). The three members of this receptor family, TrkA, TrkB, and TrkC, which are encoded by the genes NTRK1, NTRK2, and NTRK3, are located on the human chromosomes 1q23.1, 9q21.33, and 15q25.3, respectively (Gambella *et al.* 2020). These three receptor family members each possess an intracellular kinase domain, transmembrane sections, and extracellular binding domains (Gambella *et al.* 2020). Each receptor has a preferred ligand despite its high homology. Neurotrophins 4 and 5 bind to NTRK2 together with the brain-derived neurotrophic factor (BDNF), whereas neurotrophin 3 binds to both NTRK3 and NTRK1 along with the nerve growth factor (Manea *et al.* 2022). These fusion proteins might activate the RAS/MAPK/ERK, PI3K, and PLCgamma signalling pathways and stimulate the development of cancer cells (Gambella *et al.* 2020). When NTRK gene alterations take place, both neurogenic and non-neurogenic cells can undergo carcinogenesis. Although these genetic alterations are used as prognostic indicators for novel targeted therapies, solid tumours only occasionally exhibit them (Manea *et al.* 2022). TRK overexpression in neuroblastoma, basal-cell carcinomas, breast, lung, and other cancers has been reported. In BC models, overexpression of TrkA boosted tumour-cell proliferation, migration, and invasion (Cocco *et al.* 2018, Lange and Lo 2018, Wang *et al.* 2019, Hechtman 2022). Notably, 90–100% of mammary analogue secretory BCs have NTRK gene fusions (Doebele *et al.* 2020). Identification of NTRK fusions is therefore crucial for therapeutic administration (Sorber *et al.* 2022).

In this research, we sought to thoroughly assess the prognostic potential of the NTRK family of genes as biomarkers for BC. To achieve this, we utilized a range of bioinformatic tools and databases, allowing us to analyse their significance in predicting disease outcomes and tailoring treatment strategies. Through our investigation, we aimed to enhance the understanding of the role that NTRK family alterations may play in the progression of BC.

## 2 Methods

**Figure 1.**
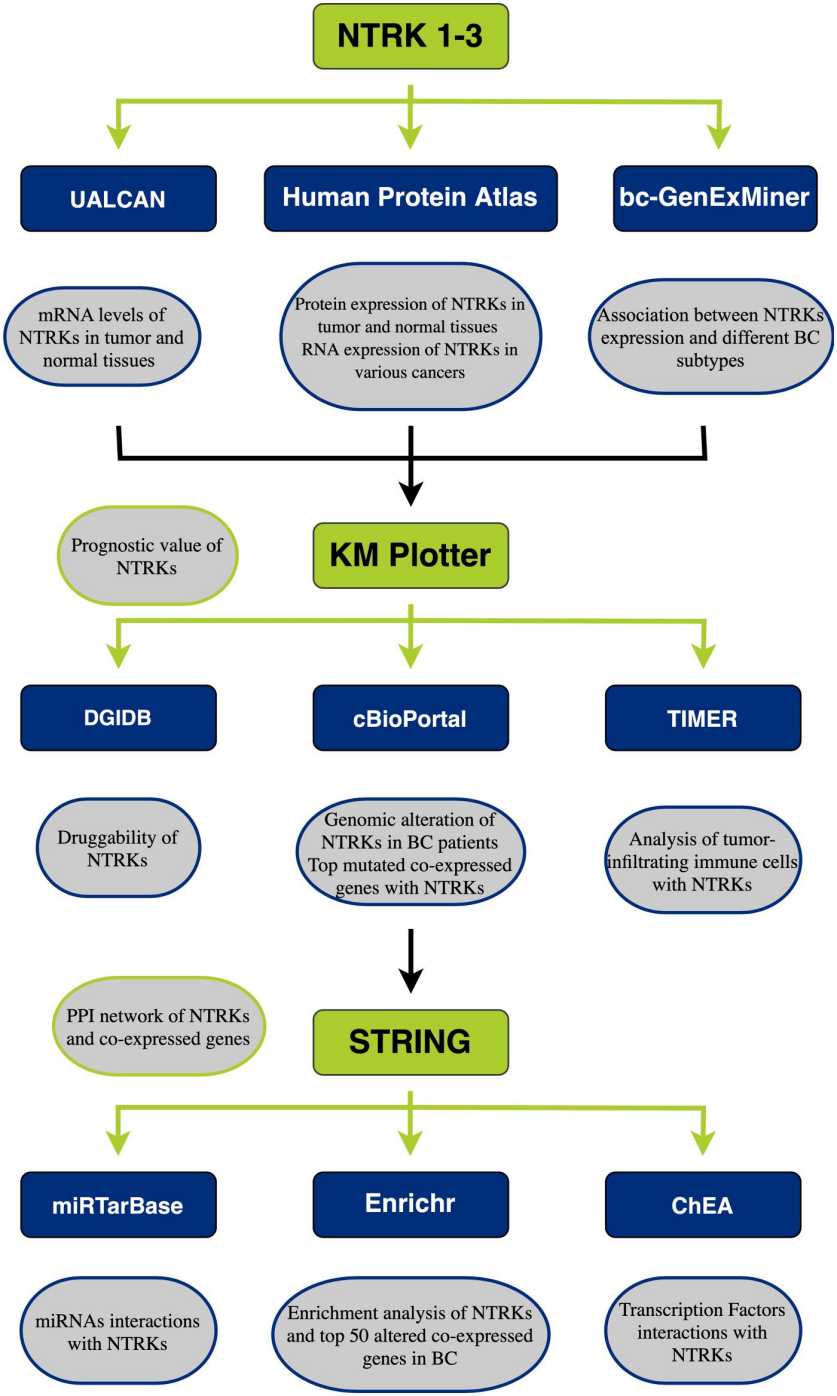
Study design flow chart.

All the data collected for conducting this study were from online databases retrieved from April to May 2024. The study methodology is summarized in Fig. 1.

### 2.1 bc-GenExMiner

The bcGenExMiner v5.0 is a web server (https://bcgenex.ico.unicancer.fr) that comprises modules for expression (Jézéquel *et al.* 2021), prognosis (Jézéquel *et al.* 2012), and correlation analysis (Jézéquel *et al.* 2013). The present study utilized the DNA microarray data of BC patients and the “expression” module of the bcGenExMiner to assess the expression of the NTRK family in accordance with the Scarff-Bloom-Richardson (SBR) grade and intrinsic molecular subtypes ascertained via the Prediction Analysis of Microarray 50 (PAM50) test. In addition, the correlation between the clinicopathological data of BC patients and the mRNA expression of NTRKs was examined. Welch’s *t*-test and Dunnett–Tukey’s Kramer’s test were utilized to calculate the *P*-value. *P* < .05 was considered to be the threshold for significance.

### 2.2 UALCAN

The UALCAN database (https://ualcan.path.uab.edu) analyses omics data from the Cancer Genome Atlas Program (TCGA) and MET500 databases that are relevant to cancer (Chandrashekar *et al.* 2022). The analysis of NTRK mRNA expression in both BC and normal tissues was performed utilizing the “TCGA gene analysis” module of the UALCAN database. The statistical analyses between groups were conducted using the Student’s *t*-test. A *P*-value less than .05 was deemed to indicate statistical significance.

### 2.3 Kaplan–Meier plotter

The online database utilized to analyse the prognostic significance of the NTRK family members in BC patients was the Kaplan–Meier plotter (https://kmplot.com), which contained survival data and gene expression profiles of cancer patients (Győrffy 2021). To determine recurrence-free survival (RFS) and overall survival (OS), samples were divided into high- and low-expression groups according to the median gene expression. In addition to the hazard ratios (HR), the 95% confidence interval (CI) and log-rank *P*-value were calculated. *P*-values less than .05 were regarded as statistically significant.

### 2.4 Human Protein Atlas

The Human Protein Atlas (https://proteinatlas.org), an online platform that presents immunohistochemistry-based expression data, illustrates the distribution and expression of a minimum of 576 immunohistochemical (IHC) staining maps for the following 20 tumour tissues, 47 cell lines, 44 human normal tissues, and 12 blood cells. The inclusion of 216 tumour tissues and 144 tissues from 144 distinct individuals ensured that the staining results were entirely representative (Wu *et al.* 2020). Using immunohistochemistry images, the protein expression of various NTRK family members in normal and BC tissues was compared. Furthermore, to compare and visualize the RNA expression of NTRKs in multiple cancers, RNA-seq data from 17 cancer types, reported as the median number of Fragments Per Kilobase of exon per Million reads (FPKM) and generated by TCGA, were retrieved from the Human Protein Atlas.

### 2.5 cBioPortal

A comprehensive online repository for presenting and evaluating multivariate cancer genomics data, cBioPortal (https://cbioportal.org), serves as a vital resource. In order to examine genomic profile changes—such as mutations, putative copy number alterations (CNAs) derived from genomic identification of significant targets in cancer (GISTIC), and mRNA expression *Z* scores (microarray)—the 1108-sample breast invasive carcinoma (TCGA, Firehose Legacy) dataset was utilized (de Bruijn *et al.* 2023). Additionally, from the co-expression module of cBioPortal, co-expressed genes related to each NTRK were retrieved; then, common genes between all NTRK family members were determined by Venn diagram, and the mutation percentage of co-expressed genes in BC was checked through the OncoPrint module of cBioPortal. The top 50 of them were selected as the frequently mutated co-expressed genes of NTRKs in BC. A *P*-value less than .05 was regarded as statistically significant.

### 2.6 STRING

By employing the STRING (https://string-db.org) database (Szklarczyk *et al.* 2019), which predicts protein–protein interactions (PPIs) through various sources, and Cytoscape (version 3.10.01) (Shannon *et al.* 2003), the PPIs network between NTRKs and the top 50 co-expressed genes was constructed with a confidence score >0.4.

### 2.7 Enrichr

Enrichr, accessible at https://maayanlab.cloud/Enrichr, is an online enrichment analysis tool (Kuleshov *et al.* 2016). Gene Ontology (GO) functional annotation [GO terms including Molecular Function (MF), Cellular Component (CC), and Biological Process (BP)] and Kyoto Encyclopedia of Genes and Genomes (KEGG) pathway enrichment analysis, Transcription Factor (TF) analysis utilizing the Chip Enrichment Analysis (ChEA) database, and miRNA prediction utilizing the miRTarBase of NTRKs and co-expressed proteins were all accomplished by Enrichr. The ggplot2 R package was employed to generate the KEGG analysis figure. *P*-value <.05 was determined as statistically significant.

### 2.8 TIMER

The Tumour IMmune Estimation Resource (TIMER 2.0), which can be accessed at https://timer.cistrome.org, is an encompassing database designed to systematically evaluate the clinical impact of immune cell infiltration across a wide range of cancer types (Li *et al.* 2020). The correlation between NTRKs expression and immune infiltrating cells, including B cells, CD8+ T cells, CD4+ T cells, macrophages, neutrophils, and Dendritic Cells (DCs), was investigated in the present study using the “gene” module of TIMER and the purity-corrected partial spearman method. *P*-values less than .05 considered to be statistically significant.

### 2.9 Drug–gene interactions database

The druggability of NTRK family members was investigated using drug–gene interactions database (DGIDB) (ver 5.0, https://dgidb.org) (Cannon *et al.* 2024). The top 10 available and approved drugs for NTRK1-3 were sorted according to interaction scores and reported.

## 3 Results

### 3.1 Association of mRNA expression level of NTRKs and BC subtypes and grades

**Figure 2.**
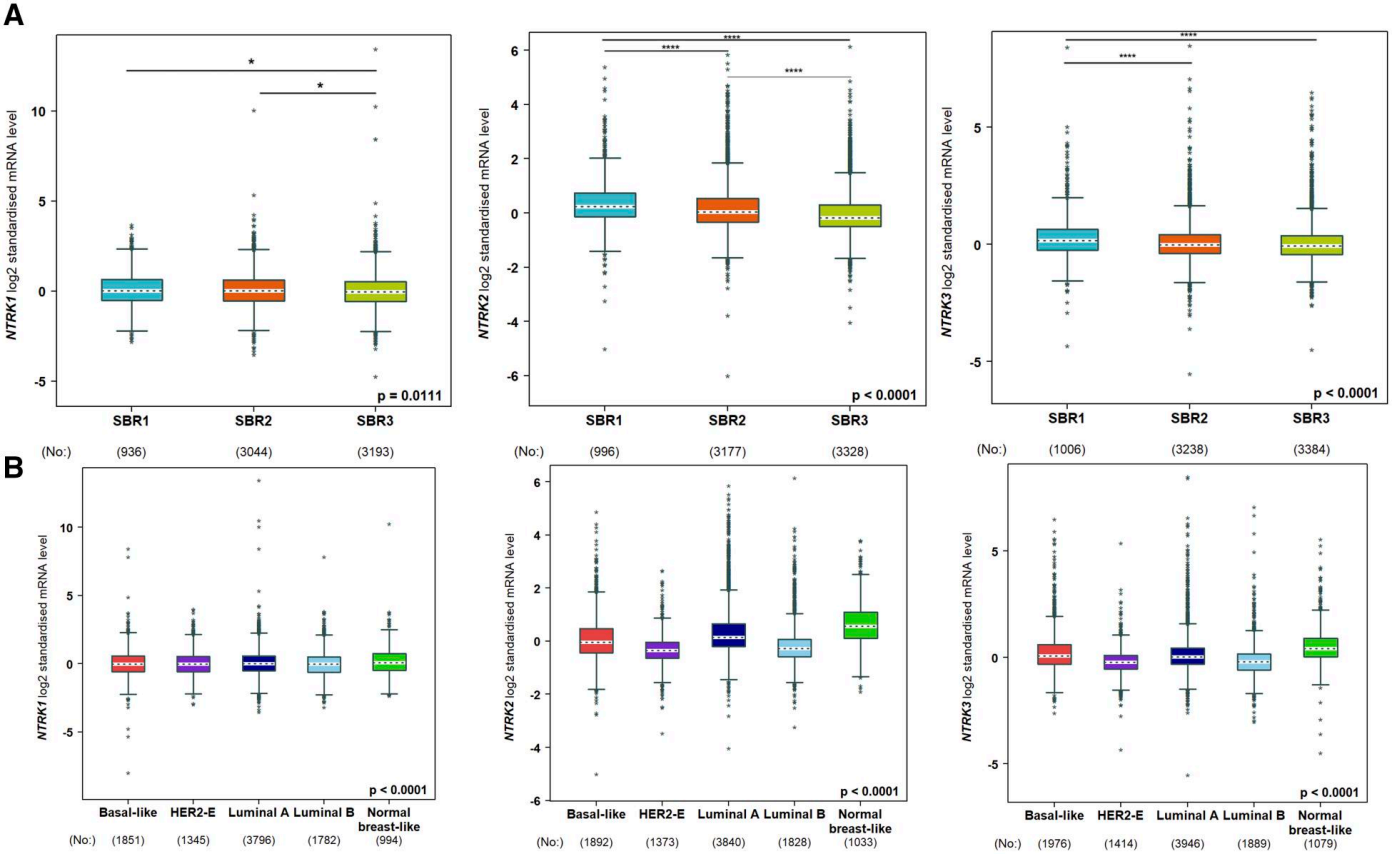
Association between NTRKs expression and SBR grade status (A) and PAM50 subtype (B) of BC patients (bc-GenExMiner v5.0). Using Welch’s tests and Dunnett–Tukey–Kramer’s test, the difference in mRNA expression between groups was assessed. The box plot of the NTRK expression goes from the lower quartile (*Q*1) to the upper quartile (*Q*3), and the median is marked with a horizontal dotted line. Whiskers are lines at the bottom and the top of the box representing the distance between the quartiles and 1.5 times the interquartile range. The number of patients is represented in brackets. *P <* *.05, **P <* *.01, ***P <* *.001, ****P <* *.0001.

The present study used bcGenExMiner v5.0 to assess the variation in NTRK mRNA expression levels among cohorts of BC patients, which were subdivided based on clinicopathological characteristics (Table 1), SBR grade status, and PAM50 subtypes. Clinicopathological data of the BC patients showed that both NTRK1 and NTRK2 were significantly upregulated in estrogen receptor-positive (ER+) patients, respectively (*P*-value: .0132 and <.0001). Likewise, in the case of PR, NTRK1 and NTRK2 were significantly upregulated in progesterone receptor+ (PR+) patients, respectively (*P*-value: .0005 and <.0001). NTRK2 and NTRK3 were notably upregulated in negative HER2 patients, respectively (*P*-value <.001 and .0001). Additionally, NTRK3 was upregulated significantly in triple-negative breast cancer (TNBC) patients (*P*-value: .0279). Eventually, NTRK1 and NTRK3 were upregulated considerably in Basal-like BC patients (*P*-value: .0019 and .0032, respectively). Regarding the SBR grade criterion, patients with primary SBR grade tended to express higher mRNA levels of NTRKs 1–3; additionally, patients with advanced SBR grade expressed lower levels of NTRKs 1/2 (*P*-value <.05) (Fig. 2A). In the case of PAM50 subtypes, the NTRK2/3 expression was decreased in HER2 patients compared to NTRK1 expression. Similarly, the expression of both NTRK2/3 declined compared to NTRK1 in Luminal B patients. However, patients with Luminal A showed high expression of the NTRK2 in comparison to other members of NTRKs. Also, patients with normal breast-like subtype expressed higher values of NTRK2/3 than NTRK1 (*P*-value <.05) (Fig. 2B).

**Table 1.** The correlation between mRNA levels of NTRKs and clinicopathological features of BC patients (retrieved from Bc-GenExminer).

Criteria	NTRK1			NTRK2			NTRK3		
	No.	mRNA	*P*-value^a^	No.	mRNA	*P*-value^a^	No.	mRNA	*P*-value^a^
Age									
≤51	267	–	.2424	267	–	.6454	267	↑	**.0005** *
>51	476	–		476	–		476	–	
Nodal status									
(−)	332	–	.8408	332	–	.8933	332	–	.7898
(+)	358	–		358	–		358	–	
ER (IHC)									
(−)	187	–	**.0132** *	187	–	**<.0001** *	187	–	.5434
(+)	530	↑		530	↑		530	–	
PR (IHC)									
(−)	243	–	**.0005** *	243	–	**<.0001** *	243	–	.4345
(+)	470	↑		470	↑		470	–	
HER2 (IHC)									
(−)	396	–	.106	396	↑	**<.0001** *	396	↑	**.0001** *
(+)	109	–		109	–		109	–	
TNBC^b^									
Not	578	–	.3985	578	–	.0631	578	–	**.0279** *
TNBC	87	–		87	–		87	↑	
Basal-like BC									
Not	605	↑	**.0019** *	605	–	.1108	605	–	**.0032** *
Basal-like	136	–		136	–		136	↑	

ER, estrogen receptor, PR, progesterone receptor, HER-2, human epidermal growth factor 2, IHC, immunohistochemistry, BC, breast cancer, No, number. ↑ means upregulated, ↓ means downregulated. Bold values represent statistically significant differences between the two groups.

a *Welch’s* tests.

b TNBC, triple-negative BC.

* *P *<* *.05.

**Figure 3.**
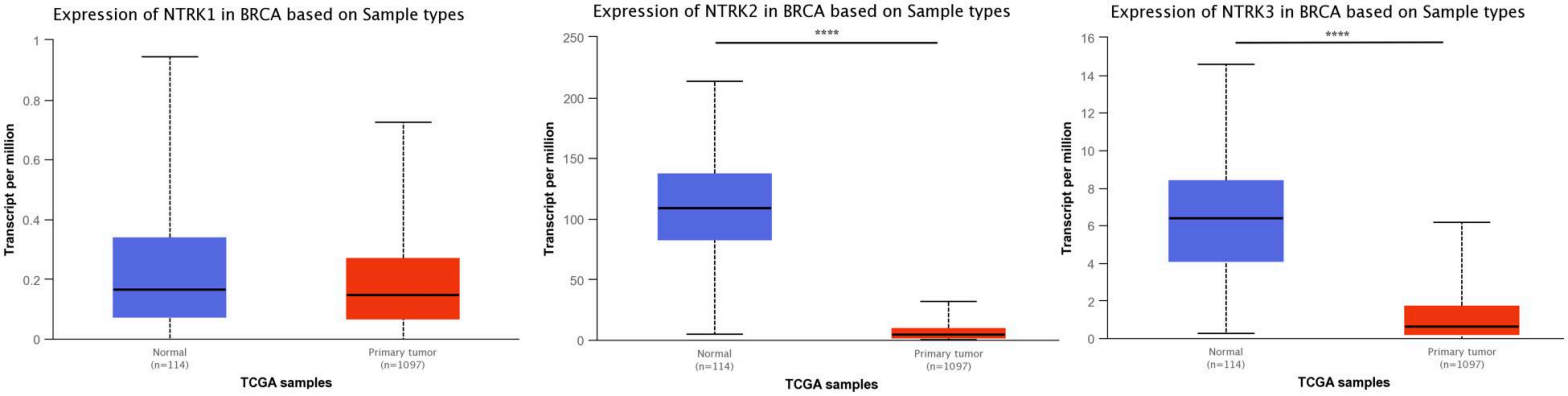
The transcription levels of NTRKs in BC and normal breast tissues (UALCAN). The Student’s *t*-test determined the statistical analysis of differential expression between groups. The *Y*-axis shows transcripts per million of each RNA molecule, and the *X*-axis represents the samples. *P <* *.05, **P <* *.01, ***P <* *.001, ****P <* *.0001.

The mRNA expression levels of NTRKs between primary tumour and normal tissues in BC patients were assessed using UALCAN. The mRNA expression levels of NTRK2/3 were significantly (*P*-value <.0001) downregulated in tumour samples (Fig. 3).

### 3.2 Prognostic value of mRNA expression of the NTRKs in BC patients

**Figure 4.**
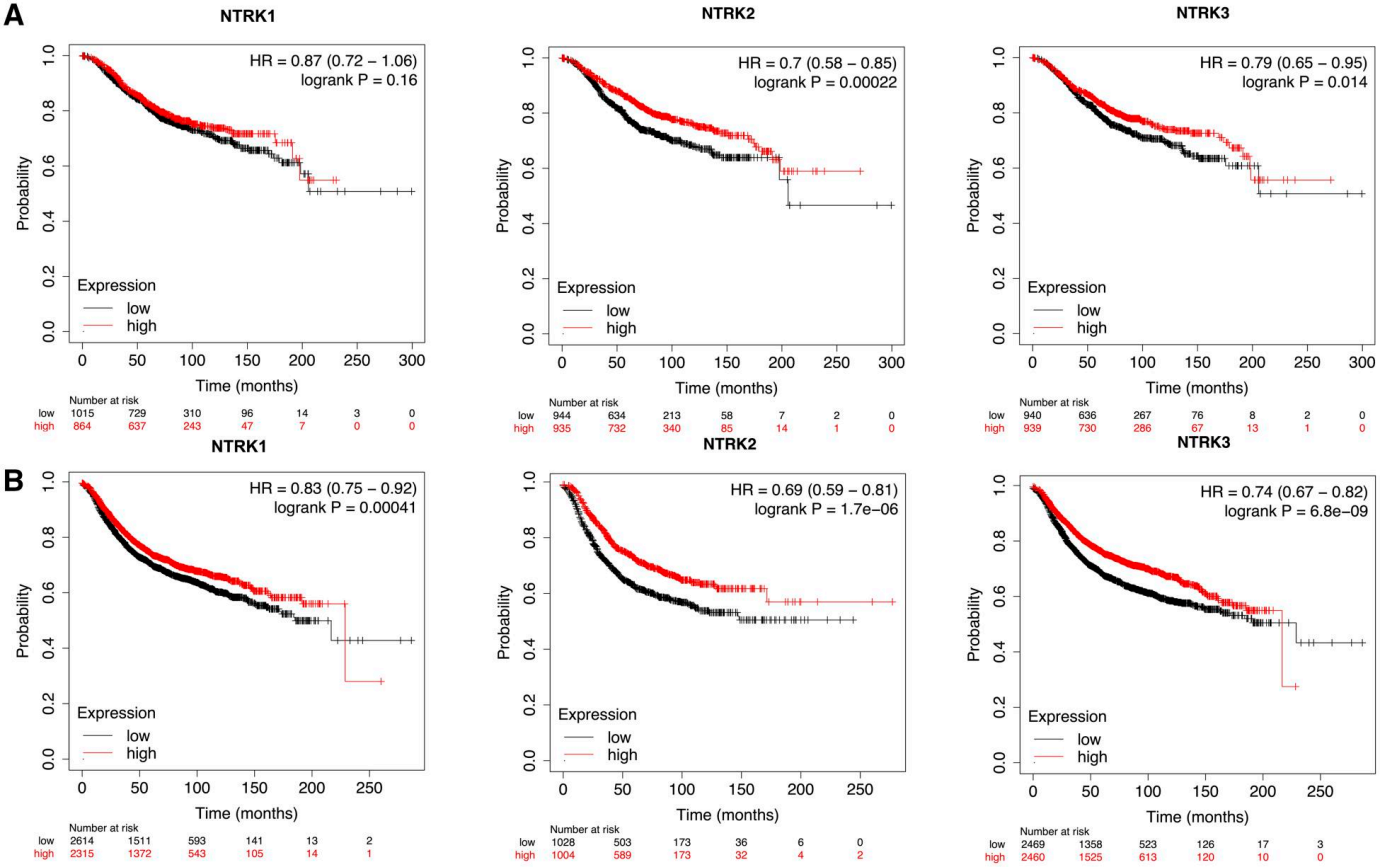
The prognostic value of the NTRKs mRNA expression (Kaplan–Meier plotter). The association of mRNA expression of NTRK family with OS (A) and RFS (B) in BC patients. Horizontal lines represent the survival curves of the patient groups with values higher and lower than the median expression levels in the target genes, respectively. The confidence intervals are represented in brackets. HR, hazard ratio.

The KM plotter was employed to evaluate the prognostic value of NTRKs among individuals with BC. The corresponding graphs indicated that elevated expression of NTRK2 and NTRK3 significantly correlated with improved OS (*P*-value <.0001 and .014, respectively). Furthermore, BC patients whose NTRK1–3 mRNA levels had risen had a substantially positive correlation with RFS (*P*-value .0001). The KM curves of NTRKs, where mRNA expression levels are substantially associated with OS and RFS, are depicted in Fig. 4.

**Figure 5.**
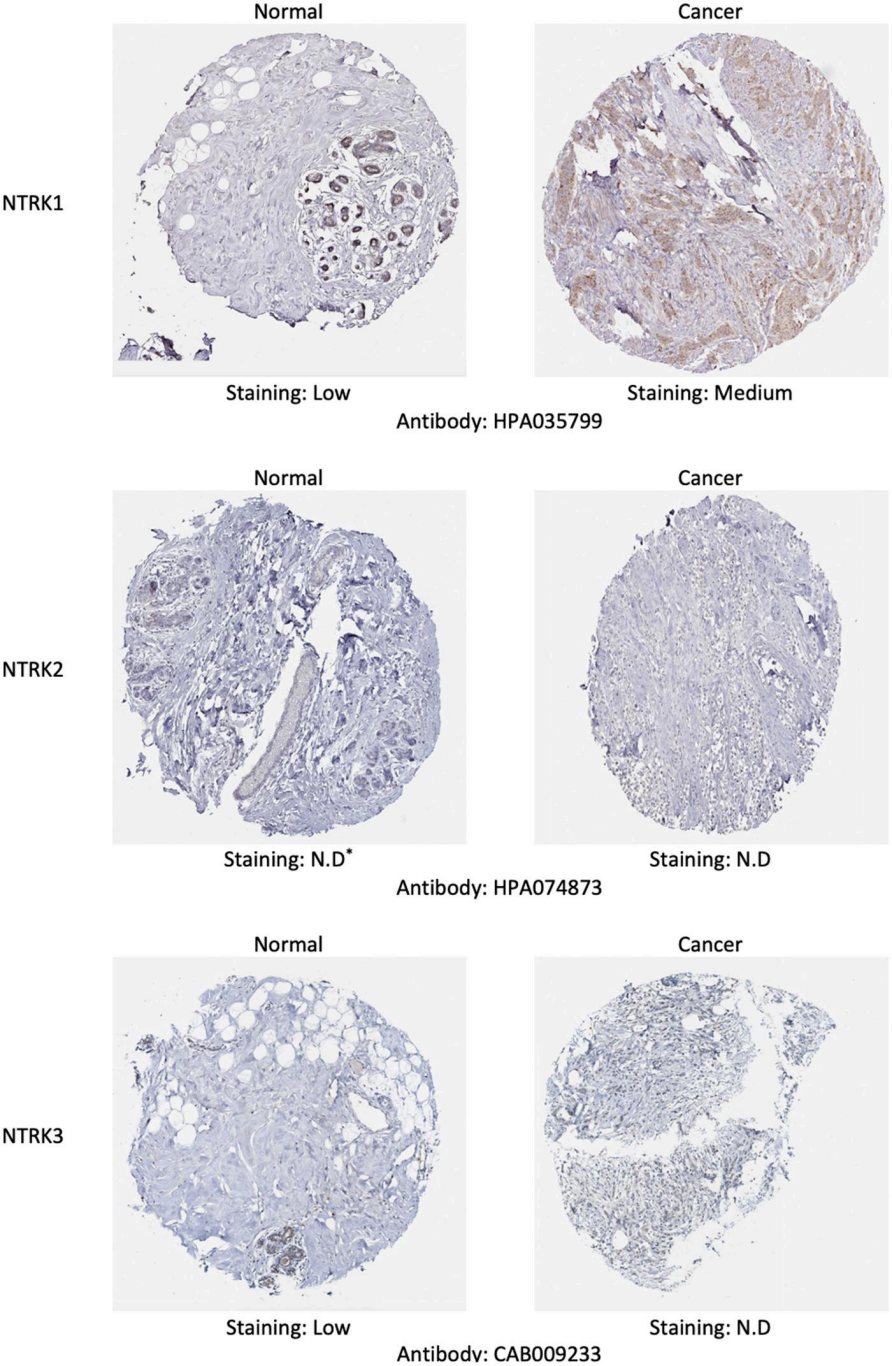
Representative immunohistochemistry images of distinct *NTRK* family members in HNSCC tissues and normal tissues (Human Protein Atlas Database). *N.D: not detected.

**Figure 6.**
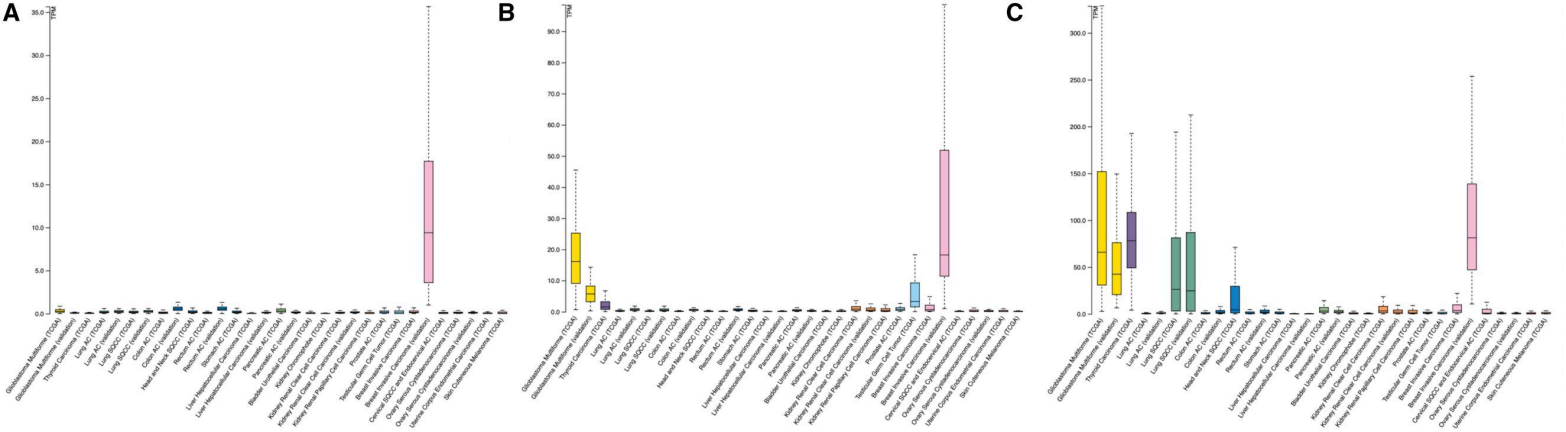
Comparison of NTRKs RNA expression in 17 different cancers according to TCGA and validation data (retrieved from Human Protein Atlas). (A) NTRK1; (B) NTRK2; (C) NTRK3. Normal distribution across the dataset is visualized with box plots, shown as median and 25th and 75th percentiles. TPM: transcript per million.

Based on IHC findings, NTRK1 was overexpressed in tumoural tissue of the BC compared to non-tumoural tissue, whereas NTRK3 was overexpressed in non-cancerous tissues. No expression was detected for NTRK2 in both normal and tumour tissues (Fig. 5). Comparison of RNA expression of NTRKs in various cancers showed that NTRK1 had a median expression of 0.2 and 9.4 TPM according to TCGA and validation methods, respectively, in the case of invasive BC. Also, NTRK2 expressed a median of 3.6 and 81.2 TPM according to TCGA and validation methods in the case of Invasive BC, respectively. Additionally, NTRK3 in Invasive BC showed 0.7 and 18.2 TPM based on TCGA and validation methods, respectively (Fig. 6).

### 3.3 Genomic alterations and GO enrichment analysis of NTRKs in BC patients

**Figure 7.**
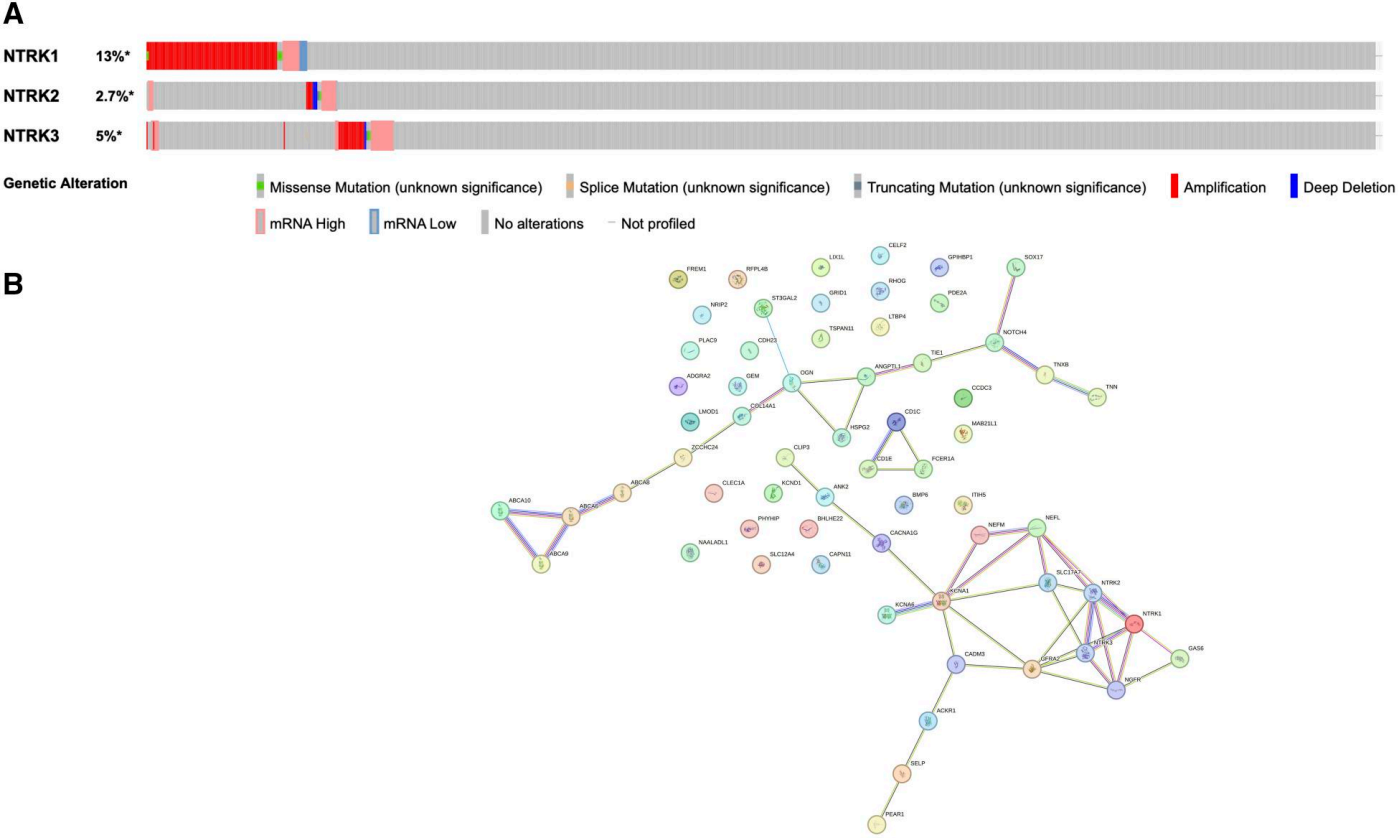
(A) Genomic alteration of NTRKs in BC patients (cBioPortal). Alteration observed in 221 (20%) out of 1101 sequenced BC patients. (B) PPI network of NTRKs and the 50 most frequently altered co-expressed genes in BC (STRING).

The cBioPortal database was employed to analyse genomic variations of the NTRKs. The findings revealed that 221 (20%) of 1101 BC patients exhibited mutations in the NTRK genes (Fig. 7A). NTRK1 was consequently mutated in 13% of patients with BC. Importantly, NTRK2 was the most conserved NTRK, and our findings revealed that it was mutated in 3% of BC cases. STRING plugin of Cytoscape was employed to map and visualize the top 50 co-expressed with NTRKs and exhibited the highest frequency of mutation in BC (Fig. 7B). The mutation percentages of the top 10 most frequently mutated co-expressing genes with NTRK family members with BC are detailed in Supplementary Table S1.

### 3.4 Enrichment analysis of NTRKs and top 50 co-expressed genes

**Figure 8.**
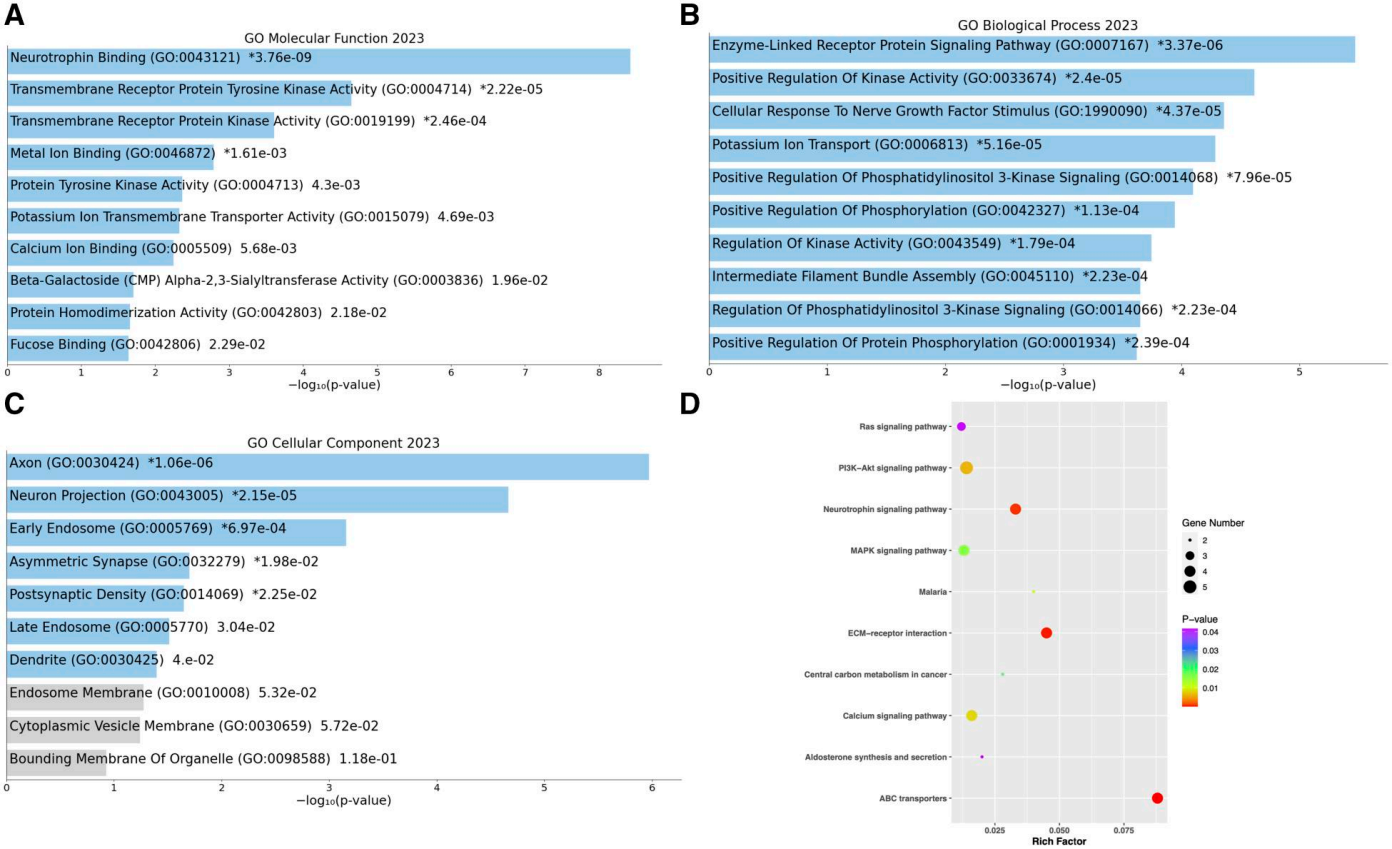
Study design flow chart. Enrichment analysis of NTRKs and top 50 altered co-expressed genes in BC. Top 10 significantly enriched GO terms in molecular functions (A), biological processes (B), and cellular components (C). The *x*-axis shows the −log10 (*P*-value), and the *y*-axis shows the GO terms, including molecular functions, biological processes, and cellular components. KEGG enrichment scatter plots of NTRKs and co-expressed genes (D). The *Y*-axis shows the KEGG pathway terms, and the *X*-axis shows the rich factor. A colour scale represents the *P*-value. A value of *P *< .05 was defined as statistically significant. Rich factor is defined as the ratio of the number of any (statistically) significant genes annotated in each specific pathway to the total number of genes annotated in that pathway.

To ascertain the pathways and roles of NTRKs, as well as their frequently altered adjacent genes, the Enrichr database was applied. NTRKs and their co-expressed genes exhibited neurotrophin binding, transmembrane receptor protein tyrosine kinase activity, and transmembrane receptor protein kinase activity as the three most significant MFs (Fig. 8A). The three most notable enriched BPs for NTRKs and their co-expressed genes were the enzyme-linked receptor protein signalling pathway, positive regulation of kinase activity, and cellular response to nerve growth factor stimulus (Fig. 8B). In case of CC, they mostly enriched as Axon, neuron projection and early endosome (Fig. 8C). NTRKs and their co-expressed genes were most frequently enriched in the ABC transporters, ECM receptor interaction and neurotrophin signalling pathway, as determined by KEGG pathway analysis (Fig. 8D).

### 3.5 TF and miRNA correlation with NTRKs

TFs and miRNAs potentially regulating NTRKs were retrieved from the ChEA and miRTarBase databases and summarized in Tables 2 and 3, respectively. Furthermore, the prognostic value of the resulting TFs and miRNAs was assessed using a KM plotter in BC. The lower expression levels of the TFs, EZH2, SUZ12 and higher expression of BACH1 and MTF2 were found to be significantly associated with better OS and prognosis in BC patients (Supplementary Fig. S1). KM curves revealed that the low expression of miR-509, miR-345, miR-617, miR-581, and miR-198 was remarkably correlated with shorter OS (all *P *<* *.05) and a worse prognosis; on the contrary, the higher expression of miR-485 showed better OS and prognosis in BC patients (Supplementary Fig. S2).

**Table 2.** The most significant TFs associating with NTRKs using ChEA database.

TF	Genes	*P*-value
EED	NTRK1, NTRK2, NTRK3	3.08E−05
SUZ12	NTRK1, NTRK2, NTRK3	1.05E−04
JARID2	NTRK1, NTRK2, NTRK3	1.46E−04
BACH1	NTRK1, NTRK2, NTRK3	1.78E−04
MTF2	NTRK1, NTRK2, NTRK3	.0019
PHC1	NTRK2, NTRK3	.0035
KLF1	NTRK2, NTRK3	.0054
TP53	NTRK2, NTRK3	.006
RNF2	NTRK2, NTRK3	.0082
EZH2	NTRK2, NTRK3	.0082

**Table 3.** The most significant miRNAs regulating NTRKs using miRTarBase database.

miRNA	Genes	*P*-value
hsa-miR-509-3p	NTRK3	.0061
hsa-miR-345-5p	NTRK3	.0076
hsa-miR-617	NTRK3	.008
hsa-miR-581	NTRK2	.0085
hsa-miR-145-3p	NTRK2	.0089
hsa-miR-485-3p	NTRK3	.01
hsa-miR-198	NTRK3	.011
hsa-miR-151a-3p	NTRK3	.0119
hsa-miR-384	NTRK3	.015
hsa-miR-4776-3p	NTRK3	.016

### 3.6 Correlation between NTRKs and immune cell infiltration in BC

**Figure 9.**
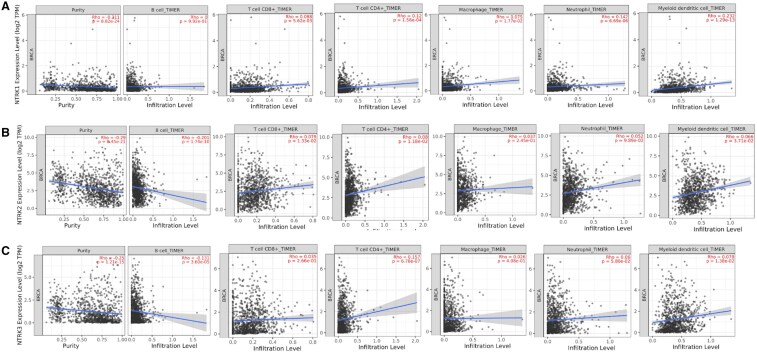
The relationship between the expression of NTRKs and the expression of tumour-infiltrating immune cells in BC from TIMER2.0. The correlation of NTRK1 (A), NTRK2 (B), and NTRK3 (C) and the expression of immune cells in BC. The partial Spearman’s correlation was used in TIMER database analysis. NTRK1 expression showed positive correlation with infiltrating levels of CD4+ T cells, neutrophil and dendritic cells. NTRK2 was negatively correlated with infiltration levels of B cell. NTRK3 had positive and negative correlation with infiltrating levels of CD4+ T cell and B cell, respectively. Positive correlation: *P*-value <.05, *ρ* > 0; negative correlation: *P*-value <.05, *ρ* < 0; not significant: *P*-value >.05.

We applied the TIMER database to evaluate the correlation between NTRK expression and immune cell infiltration (B cells, CD8+ T cell, CD4+ T cell, macrophage, neutrophil, and DCs) in BC. The results indicated that the expression of NTRK 1 (Fig. 9A) was positively correlated with the infiltration of CD4+ T cells, neutrophils and DCs (*P*-value <.05, *ρ *>* *0). Moreover, we found that the expression of NTRK2 was negatively correlated with infiltration levels of B cells (*P*-value <.05, *ρ *<* *0) in BC (Fig. 9B). Also, NTRK3 expression (Fig. 9C) was positively associated with infiltration abundances of CD4+ T cell (*P*-value <.05, *ρ *>* *0). At the same time, it showed a negative correlation with infiltration levels of B cells (*P*-value <.05, *ρ *<* *0).

### 3.7 Predicted drug candidates

The nominated drugs potentially targeting NTRK1-3 were acquired using the DGIdb database. Our results showed that larotrectinib, the approved inhibitor of TRKs, had the highest potential to inhibit all three NTRK1, NTRK2, and NTRK3 genes. Secondly, Prasugrel, an antiplatelet agent, could target NTRK1 efficiently. Lastly, Larotrectinib Sulfate (NTRK inhibitor) presented an inhibitory interaction with NTRK3 and NTRK2 genes (Table 4).

**Table 4.** Prediction of NTRK1–3 genes’ druggability by the DGIDB database.

Drug	Targeted gene	Interaction score
Larotrectinib	NTRK3	1.83
Larotrectinib	NTRK2	1.36
Larotrectinib	NTRK1	1.078
Prasugrel	NTRK1	0.47
Larotrectinib sulfate	NTRK3	0.40
Larotrectinib sulfate	NTRK2	0.36
Cenegermin-bkbj	NTRK3	0.30
Corticotropin	NTRK2	0.27
Cenegermin-bkbj	NTRK2	0.27
Vitamin B6	NTRK1	0.23

## 4 Discussion

Prognosis in cancers, precisely BC, is of great importance, but the ongoing challenges caused by this complex disease on global scale are ever-present. Despite significant advances in cancer research, BC remains a significant public health concern worldwide, affecting millions of people (Obeagu and Obeagu 2024). Due to its aggressive nature, this cancer is considered the primary cause of cancer-related deaths among women (Saroğlu *et al.* 2024). The human neurotrophins, consisting of four ligands, play a crucial role in various cellular functions. The NTRK family, which includes NTRK1, NTRK2, and NTRK3 genes, encodes these Trk receptors and their respective ligands, affecting key signalling pathways involved in cancer progression (Hondermarck 2012, Tajbakhsh *et al.* 2017, Saroğlu *et al.* 2024). NTRK gene changes have been shown to play a role in tumorigenesis. Early detection of changes in this gene family can lead to improved patient management by the medical team (Zito Marino *et al.* 2023). Furthermore, the use of these biomarkers goes beyond traditional tests and can enhance the accuracy of diagnosis and selection of appropriate treatment for BC patients. Detection of NTRK gene fusions is feasible through multiple methods, including DNA and RNA sequencing of tumour samples, underscoring their potential clinical utility in patient management strategies. Treatment of individuals with NTRK fusion-positive cancers using first-generation TRK inhibitors like Larotrectinib or Entrectinib has shown a significant response rate exceeding 75%, irrespective of the specific histology of the tumour (Cocco *et al.* 2018). NTRK protein binding partners mainly affect tumourigenesis or survival in BC patients, particularly those with NTRK gene fusions through oncogenic mechanisms—for instance, ETV6-NTRK3, an infrequently occurring but potent oncogenic driver in secretory breast carcinoma. Activation of NTRK signalling pathways that could stimulate cell proliferation and tumourigenesis is the consequence of these gene fusions. Also, NTRK gene fusions result in the production of Trk receptors (TrkA, TrkB, TrkC); the activated TRK receptors trigger principal signalling pathways, particularly the mitogen-activated protein kinase (MAPK) and phosphoinositide 3-kinase (PI3K) pathways. In BC models, overexpression of TrkA has been shown to enhance tumour cell proliferation, migration, and invasion through these pathways. This signalling cascade is crucial for various cellular functions related to tumour progression (Cocco *et al.* 2018, Wu *et al.* 2021). Besides, nerve growth factor (NGF) and brain-derived neurotrophic factor (BDNF) are the chief ligands of TrkA and TrkB, respectively, which trigger downstream signalling pathways that stimulate cell survival and differentiation and more accurately promote tumourigenesis in BC (Bungaro and Garbo 2024).

NTRK1 and NTRK2 are implicated in both the progression of cancer and the response to therapeutic interventions, according to previously conducted investigations (Stravodimou and Voutsadakis 2022, Wang *et al.* 2024). The identification of NTRK1 and NTRK2 upregulation among patients diagnosed with ER+ BC implies that upregulation in ER+ patients may play a role in facilitating tumour survival or growth within this subtype. In patients with PR+ BC, there are notable alterations in the expression levels of both NTRK1 and NTRK2. The upregulation of both NTRK1 and NTRK2 could imply that it contributes to the progression of PR+ tumours or affects their response to hormonal therapies. Additionally, we examined the levels of NTRK2 and NTRK3 expression in patients diagnosed with BC who had tumours positive for HER2. The significant increase in NTRK2 expression indicates its possible participation in pathways vital for the survival of tumours, particularly those that are HER2-negative in BC. Activation of the NTRK2 and NTRK3 signalling pathways has been associated with tumour invasion, resistance to therapy, and the progression of numerous malignancies, including BC. Multiple malignancies have been linked to dysregulation of the NTRK3 signalling pathway, which has been shown to regulate neuronal survival and differentiation (Cocco *et al.* 2018, Regua *et al.* 2019). As the overexpression of NTRKs on hormone receptor-positive (HR+) BC was reported in studies (Hechtman 2022), we showed that NTRK1 and 2 upregulated in PR+ patients. Also, NTRK3 is upregulated in negative HER2 and TNBC patients.

High levels of NTRK2/3 expression showed a better relationship with OS and RFS in BC patients and are good prognosis markers. It was stated that NTRK2 is associated with tumour development (Wang *et al.* 2019). A study reported no statistically significant association between NTRK fusion and progression-free survival (PFS) in solid tumours (Zhu *et al.* 2022). A recent study reported a prevalence of approximately 0.7% for NTRK-positive colorectal cancer (CRC), with genetic profiling of 2519 colon and rectal tumours (Wang *et al.* 2022). The presence of the TrkA receptor is detected in BC cell lines and tissues, showing higher levels compared to the normal breast epithelium (Lagadec *et al.* 2009). In BC cells, the activation of TrkA sends signals through the MAPK pathway in a manner that aligns with the normal functioning of the receptor (Bradshaw *et al.* 2013). Conversely, the present investigation demonstrated that elevated levels of NTRK2/3 had better prognosis potential in BC patients. The genomic alteration of NTRKs showed that NTRK1 mutated in 13% of BC patients (according to TCGA data). However, it did not show any changes in the expression of the mentioned gene, which could be accounted for the type of occurred mutation (mostly missense), which unexceptionally does not affect the mRNA expression level (Fig. 7A). Furthermore, several other reasons might explain this, including rare occurrence of the NTRK fusions in BC (O’Haire *et al.* 2023), mutual exclusivity with other oncogenic drivers (Westphalen *et al.* 2021) and tumour heterogeneity (Xulu *et al.* 2023).

The results of this study showed that there is a correlation between the expression of NTRKs and the infiltration of immune cells in BC. The expression of NTRK1 was found to have a significant positive association with the infiltration of CD4+ T-cells, neutrophils and DCs. The presence of tumour-infiltrating immune cells is considered one of the most important indicators of immune system monitoring and an integral part of the complex tumour microenvironment (Domingues *et al.* 2016). CD4+ T-cells are also among the principal cells and coordinators of innate immune responses, and research that they play a role as effective anti-tumour cells. These cells directly annihilate tumour cells through cytolysis (Borst *et al.* 2018, Speiser *et al.* 2023). In the present study, the expression level of NTRK2/3 mRNA decreased significantly (*P*-value <.0001) in tumour samples (Fig. 3). In a study conducted on MKN-28 and SNU-719 Gastric Cancer (GC) cells, it was found that NTRK2 is aberrantly expressed in GC cell lines compared to normal gastric cells (Hu *et al.* 2016). Overall, our results showed that NTRK3 could be a potential biomarker of choice in BC. TrkC is involved in various cell signalling pathways. MAPK and PI3K/AKT pathways are among these pathways that can play an important role in cell growth and differentiation (Okamura *et al.* 2018). Numerous recent studies have highlighted the significance of the TRK pathway in various genetic disorders, including single nucleotide variations and gene fusions found in a wide array of tumours. Notably, NTRK3 gene fusion disorders have been definitively linked to carcinogenesis (Khotskaya *et al.* 2017). The overexpression of TrkC has been documented in several cancers, such as BC (Mortensen *et al.* 2021), hepatocellular carcinoma (Wang *et al.* 2023), and metastatic melanoma (Lezcano *et al.* 2018, Forschner *et al.* 2020). Furthermore, the crucial role of TrkC in regulating angiogenesis and facilitating metastasis cannot be overstated. Alterations in the expression of NTRK3 and its fusion proteins may profoundly impact processes like epithelial–mesenchymal transition (EMT), tumour growth rate, and tumourigenesis (Jin 2020).

Larotrectinib is an orally administered, specific inhibitor of NTRKs, which has remarkable anticancer efficacy regardless of the age and tumour type of the patients in NTRK fusion-positive cancers (Drilon *et al.* 2018, Scott 2019). Also, Larotrectinib sulfate is the sulfate salt form of Larotrectinib. Sulfate salt is used to enhance the drug’s stability and solubility. Our study found that all three NTRKs highly inhibited by Larotrectinib. While Larotrectinib sulfate had only prohibited NTRK2 and NTRK3, Prasugrel—a thienopyridine adenosine diphosphate (ADP) receptor antagonist for the reduction of acute myocardial infarction in individuals with acute coronary syndrome after percutaneous coronary intervention (PCI) (Metkus *et al.* 2024)—could only present inhibitory effects against NTRK1.

Our findings reveal intriguing insights into the expression patterns and prognostic implications of NTRKs in BC. Conclusively, our bioinformatic analysis underscores the prognostic significance of the NTRK family in BC and offers valuable insights that could guide the development of predictive biomarkers and therapeutic strategies. Comprehensive evaluation of NTRK in BC patients in early stages or people with a family history of BC can provide a new approach to precise and personalised medicine as an effective treatment strategy. Likewise, further studies are required to validate and confirm our findings in an *in vivo* situation.

## Supplementary Material

vbaf030_Supplementary_Data

## Data Availability

All data generated or analysed during this study are included in this published article.
