# Supplementary material for: A comprehensive bioinformatic evaluation of the NTRK family’s potential as prognostic biomarkers in breast cancer
Source: Bioinform Adv. 2025 Feb 21;5(1):vbaf030. doi: 10.1093/bioadv/vbaf030 (PMC11886811; doi:10.1093/bioadv/vbaf030)
Supplement: vbaf030_Supplementary_Data [file vbaf030_supplementary_data.zip › Proof_Supp Mat.docx]

*Supplementary materials*

**A comprehensive bioinformatic evaluation of the NTRK family's potential as prognostic biomarkers in Breast Cancer**

Ramtin Mohammadi^1,2^,Mohsen Ghiasi^3,◊^, Saber Mehdizadeh^4,◊^, Javad Mohammadi^1,5^, Shahla Mohammad Ganji^1*^

^1^Department of Molecular Medicine, Medical Biotechnology Institute, National Institute of Genetic Engineering and Biotechnology (NIGEB), Tehran, Iran.

^2^Medical Biotechnology and Bioinformatics Research Group (MBBRG), Universal Scientific Education and Research Network (USERN), Tehran, Iran.

^3^Rajaie Cardiovascular Medical and Research Center, Iran University of Medical Sciences, Tehran, Iran.

^4^Department of Immunology, School of Medicine, Mazandaran University of Medical Sciences, Sari, Iran.

^5^Department of Life Science Engineering, Faculty of New Sciences and Technologies, University of Tehran, Tehran, Iran.

***Corresponding author.** Department of Molecular Medicine, Medical Biotechnology Institute, National Institute of Genetic Engineering and Biotechnology (NIGEB), Tehran, Iran. **E-mail:** [shahla@nigeb.ac.ir](mailto:shahla@nigeb.ac.ir)

| 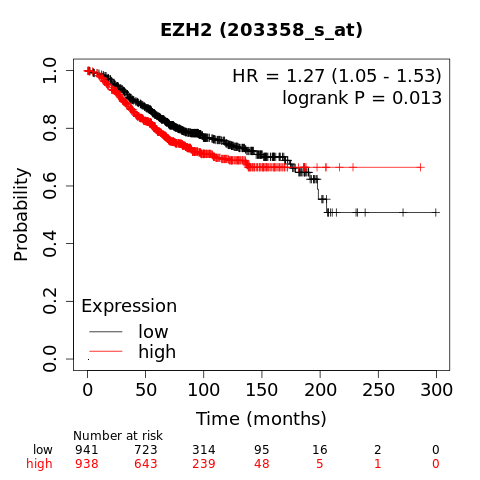 | 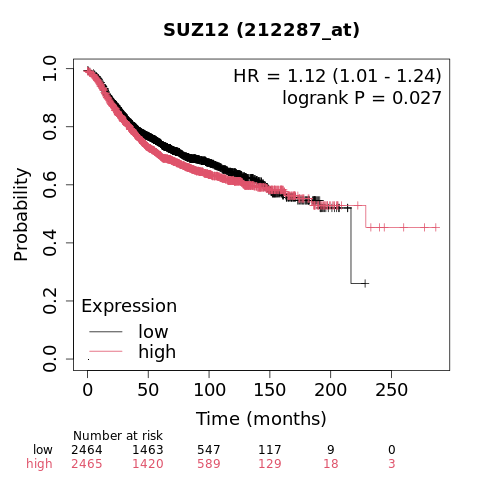 |
| --- | --- |
| 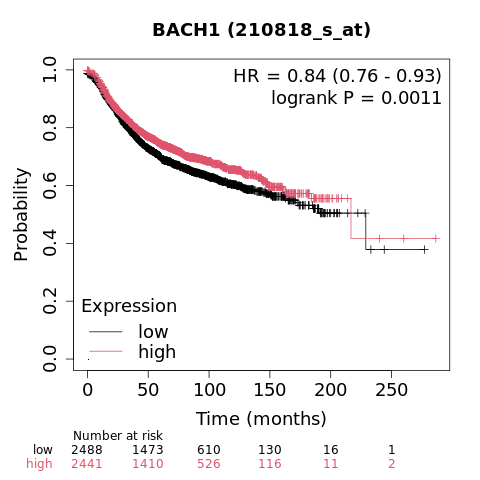 | 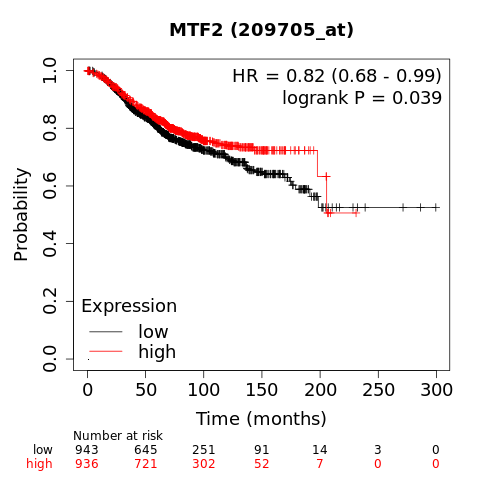 |
|  |  |

**Fig S1.** The prognostic value of the TFs regulating all three NTRKs (Kaplan-Meier plotter). The association of mRNA expression of EZH2, SUZ12, BACH1 and MTF2 with OS in BC patients. A value of P < 0.05 was defined as significant. The confidence intervals are represented in brackets. Red: high expression level; black: low expression level. HR, hazard ratio.

| 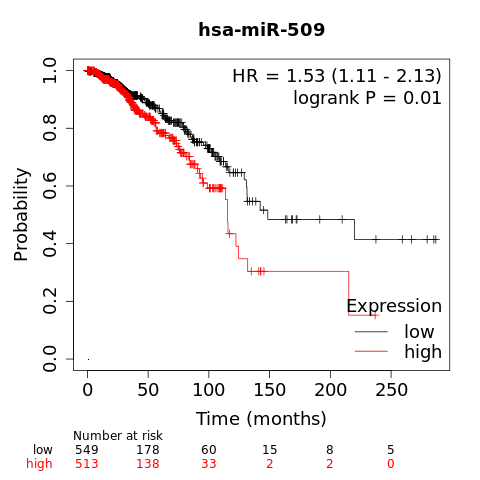 | 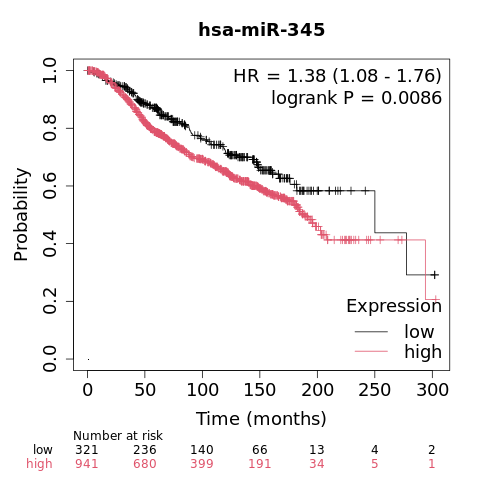 |
| --- | --- |
| 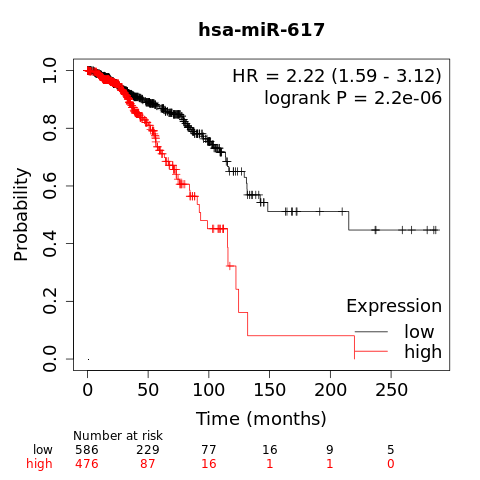 | 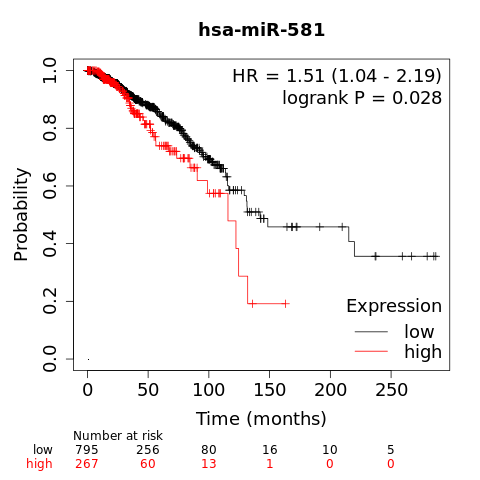 |
| 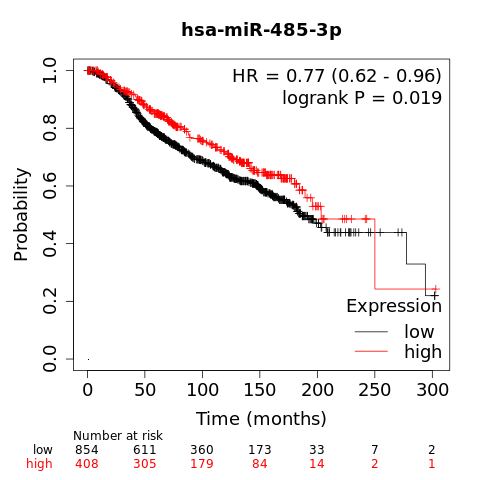 | 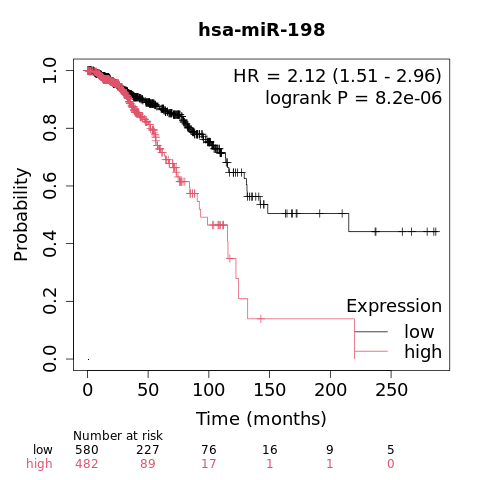 |

**Fig. S2.** The prognostic value of the miRNAs regulating NTRKs (KMplotter). The association of miR-509, miR-345, miR-617, miR-581, miR-485-3p and miR-198 expression with OS in BC patients. A value of P < 0.05 was defined as significant. The confidence intervals are represented in brackets. Red: high expression level; black: low expression level. HR, hazard ratio.

**Table S1.** The top 10 frequently mutated (%) genes with NTRKs in breast cancer.

| Gene symbol | Mutation % |
| --- | --- |
| COL14A1 | 21 |
| ADGRA2 | 17 |
| MAO1LC3C | 15 |
| GPIHBP1 | 15 |
| GEM | 15 |
| LIX1L | 14 |
| SELP | 13 |
| PEAR1 | 13 |
| FCER1A | 13 |
| LMOD1 | 13 |
| CD1E | 13 |
